# Beyond the Funding: Barriers and Facilitators to VA Medical Foster Home Program Sustainment

**DOI:** 10.1093/geroni/igaf122.3548

**Published:** 2025-12-31

**Authors:** Chelsea Manheim, Rachel Fix, Leah Haverhals

**Affiliations:** VA, Aurora, Colorado, United States; VA Health Services Research and Development, Aurora, Colorado, United States; VA Eastern Colorado Healthcare System, Aurora, Colorado, United States

## Abstract

The VA Medical Foster Home (MFH) program provides home-based long-term care for Veterans requiring nursing home level support. VA MFH coordinators recruit community-based non-VA caregivers and match them with Veterans to live in the caregiver’s home. Veterans pay caregivers directly and caregivers provide them around the clock care while caring for up to three residents. Since 2017, the VA Office of Rural Health (ORH) has funded new MFH programs in rural areas. The purpose of this abstract is to describe barriers and facilitators to MFH program sustainment identified through six interviews conducted from 2024-2025 with MFH coordinators from six ORH-funded MFH sites across six US states. To analyze data, we applied the REAIM implementation science framework and a team-based inductive and deductive thematic approach. Barriers to expanding reach included lack of timely identification of Veterans appropriate for MFH when space in a MFH became available. This left some caregivers with open rooms in their MFH longer than was ideal. Challenges of recruiting caregivers in rural areas emerged as a barrier to program adoption. Certain state laws acted as barriers to implementation, causing complications for MFH program growth. Veterans quality of life proved very high in MFHs, which facilitated program effectiveness. When MFH coordinators had longer tenure in their role and more experience, this facilitated maintenance and areas to target to expand and sustain programs. As the Veteran population rapidly ages demand for the MFH program will increase. These findings will inform program leadership in strategizing for effective program sustainment.

